# Preparing home health clinicians to educate caregivers of persons living with dementia

**DOI:** 10.1002/alz.087818

**Published:** 2025-01-09

**Authors:** Tammi Albrecht, Sarah Endicott, Kristen E Kehl‐Floberg, Molly Schroeder, Noelia Sayavedra, Jane Mahoney, Cynthia M. Carlsson, Art Walaszek

**Affiliations:** ^1^ Wisconsin Alzheimer’s Institute, University of Wisconsin School of Medicine and Public Health, Madison, WI USA; ^2^ University of Wisconsin School of Nursing, Madison, WI USA; ^3^ University of Wisconsin‐Madison, Madison, WI USA; ^4^ University of Wisconsin‐Madison Population Health Institute, Madison, WI USA; ^5^ University of Wisconsin School of Medicine and Public Health, Madison, WI USA; ^6^ School of Medicine and Public Health, University of Wisconsin‐Madison, Madison, WI USA

## Abstract

**Background:**

The majority of persons living with moderate to severe dementia live in their homes despite the challenges of increasing care needs as dementia progresses. Caregivers are not prepared to understand and manage common medical concerns, such as incontinence, dehydration, and impaired mobility. Health care clinicians need training and tools to better prepare caregivers for these responsibilities. The goal of this program was to address this need by developing an educational program for home health clinicians who treat and support persons living with moderate to severe dementia in their homes.

**Methods:**

Participants included registered nurses, physical therapists, occupational therapists, speech therapists, and social workers from two home health agencies. Trainings were held throughout a two‐year period. Outcomes measured included clinician knowledge, attitudes, and self‐efficacy gathered via surveys at baseline, immediately after training, and six‐months post‐training. Outcomes measured also included caregiver burden, caregiver self‐efficacy, and use of medical services and community resources gathered via surveys at baseline and six‐months post‐training.

**Results:**

Twenty‐one clinicians were trained; 19 completed the baseline survey and 11 completed the post‐training survey. The clinicians implemented the caregiver training with 29 caregivers; 24 completed the baseline survey and 11 completed the six‐month post‐training survey. Clinicians demonstrated improvements in knowledge and self‐efficacy post‐training. Clinicians experienced significant improvements in attitudes as measured by the Dementia Attitudes Scale post‐training, especially in comfort and familiarity with caring for persons living with dementia. Caregivers reported similar levels of caregiver burden and self‐efficiency at baseline and six‐months post‐training. Caregivers reported overall satisfaction with the training they received from home health clinicians.

**Conclusion:**

Persons living with dementia and their caregivers are impacted by changes in cognition, function, and physical health as dementia progresses. Home health clinicians are well‐positioned to help address these needs. The training provided to clinicians led to improvement in attitudes and confidence in the care of persons with dementia. Family caregivers reported a high level of satisfaction with the training; however, a small sample size and the effects of the COVID‐19 pandemic affected the ability to identify changes in caregiver burden, caregiver self‐efficacy, and use of medical and social services.

